# Selective Dye Adsorption by Zeolitic Imidazolate Framework-8 Loaded UiO-66-NH_2_

**DOI:** 10.3390/nano9091283

**Published:** 2019-09-08

**Authors:** Hao Zhang, Xiaobo Shi, Jialiang Li, Parveen Kumar, Bo Liu

**Affiliations:** 1 School of Chemistry and Chemical Engineering, Shandong University of Technology, Zibo 255000, China; 2 Laboratory of Functional Molecules and Materials, School of Physics and Optoelectronic Engineering, Shandong University of Technology, Zibo 255000, China; 3 Department of Physics, Henan Finance University, Zhengzhou 450046, China

**Keywords:** ZIF-8, UiO-66-NH_2_ composites, metal-organic frameworks, selective adsorption

## Abstract

In this study, Zeolitic Imidazolate Framework-8 (ZIF-8)-loaded UiO-66-NH_2_ was synthesized, characterized, and analyzed for its potential to efficiently remove dyes. The selective adsorption on ZIF-8-loaded UiO-66-NH_2_ or its parent MOFs (UiO-66-NH_2_ and ZIF-8) in the mixed dyes solution was explored, including anionic dye (methyl orange (MO)) and cationic dyes (methylene blue (MB) and rhodamine B (RhB)). ZIF-8-loaded UiO-66-NH_2_ displayed much better selectivity to MB than its parent MOFs. Adsorption capacity of ZIF-8-loaded UiO-66-NH_2_ (173 mg/g) toward MB was found to be 215% higher than UiO-66-NH_2_ (55 mg/g). A kinetics study based on adsorption data demonstrated that the adsorption process most closely matched with the model of pseudo-second-order kinetic and Langmuir isotherm. The adsorption was an exothermic and spontaneous physical process as revealed by the values of thermodynamic parameters. Furthermore, reusability of ZIF-8-loaded UiO-66-NH_2_ was investigated and revealed the significant regeneration efficiency in adsorption capacity for MB even after four adsorption cycles. Experimental results proved that the interaction between ZIF-8-loaded UiO-66-NH_2_ and MB was mainly affected by the mechanism, for instance, electrostatic interaction as well as π–π stacking interactions.

## 1. Introduction

Metal organic frameworks (MOFs) are three-dimensional porous crystal materials consisting of metal clusters or ions coordinated with organic ligands [1]. MOFs are the emerging highly ordered crystalline porous materials with advantages such as, for instance, large specific surface area, unconventional porosity, good chemical stability, and tunable structural and functional properties [2].

UiO-66-based composites, including polymer (polymer@UiO-66) [3], nanoparticles (NPs@UiO-66) [4], and MOF (MOF@UiO-66) [5] have become a focus of research due to the high stability of UiO-66. Owing to the interface between single MOF, the MOF@MOF structures can provide new capabilities [6] and have been widely employed for gas storage and catalysis. For instance, Ren et al. [7] synthesized MIL-101@UiO-66 nanocrystals, which were used for hydrogen storage and displayed significantly improved performance of hydrogen storage compared with parent MOF (MIL-101 or UiO-66). Zhuang et al. [8] synthesized core–shell UiO-66-NH_2_@ZIF-8 and further transformed it into Pd-UiO-66-NH_2_@ZIF-8 to achieve enhanced selective catalysis for alkene hydrogenations. Although MOF@MOF showed appreciably better performances than the parent MOFs, so far MOF@MOF has not been widely used for the removal of dye.

To date, many approaches have been developed for the elimination of dyestuff, such as adsorption [9], biodegradation [10], photocatalytic oxidation [11], and membrane separation [12]. Among them, adsorption is deemed as an effective way owing to its low cost, high efficiency, simple actions and environment-friendliness [13]. Nevertheless, the majority of the reported adsorbents exhibit several drawbacks, such as low adsorption capacity and poor selectivity, as well as difficulty in solid-liquid separation. It is essential to find desirable adsorption materials that can reduce pollutant organic dyestuff with high selectivity and adsorption capacity. The outstanding features of MOF@MOF provide an alternative way for the desirable adsorption materials to achieve selective adsorption.

ZIF-8 (Zeolitic Imidazolate Framework-8) is a metal organic framework material with a zeolite SOD (Sodalite) topology, made by Zn^2+^ ions coordinated with imidazole rings [14]. ZIF-8 has pore size between 4–5 Å and possesses a large specific surface area and good water stability as well as it contains abundant acid-base groups. Therefore, it is being used in various fields, especially for the separation of pollutants by adsorption [15,16,17,18,19]. Feng et al. [17] studied the adsorption behavior and mechanism between methyl blue (MB) and ZIF-8 in detail. Thanh et al. [18] reported a one-step synthesis of Fe-ZIF-8 and used it for the removal of Remazol Deep Black RGB dye from aqueous solutions. Zhou et al. [19] found that ZIF-8 could simultaneously remove 95.4% Cu and 80.3% norfloxacin.

In this work, we have synthesized UiO-66-NH_2_ and ZIF-8-loaded UiO-66-NH_2_. The successful combination of ZIF-8 and UiO-66-NH_2_ was characterized by X-ray diffraction (XRD), scanning electron microscopy (SEM), Fourier transform infrared (FT-IR) spectroscopy, energy-dispersive X-ray spectroscopy (EDS) elemental mapping, and transmission electron microscopy (TEM). Three typical representative dyes with different charge, including methyl orange (MO), MB, and rhodamine B (RhB) were applied to ascertain selectivity of the adsorbent. The MB adsorption capacity of ZIF-8-loaded UiO-66-NH_2_ was found to be higher than its parent MOFs (UiO-66-NH_2_ or ZIF-8). We also demonstrated the isotherm, kinetics, and thermodynamics of the MB adsorption on ZIF-8-loaded UiO-66-NH_2_ and UiO-66-NH_2_ to understand the mechanisms behind the adsorption behavior.

## 2. Materials and Methods

### 2.1. Materials

2-aminoterephthalic acid (H_2_BDC-NH_2_, 98%), Zirconium chloride (ZrCl_4_, 99.9%), and 2-methylimidazole (2-MI, 99%) were purchased from Aladdin Biochemical Technology Co., Ltd. (Shanghai, China). Zinc nitrate hexahydrate (Zn(NO_3_)_2_·6H_2_O, 99%), methanol (CH_3_OH, 99.5%), MB, MO, RhB, hydrochloric acid (HCl, 37 wt%), sodium hydroxide (NaOH, 96%), and *N*,*N*-dimethylformamide (DMF, 99.5%) were purchased from Sinopharm Chemical Reagent Co., Ltd. (Shanghai, China). All solvents and reagents were of analytical reagent (AR) grade and used as received.

### 2.2. Synthesis of UiO-66-NH_2_

The solvothermal method was used to prepare UiO-66-NH_2_, according to the procedure reported in the previous literature [20]. ZrCl_4_ (5 mmol) was dispersed in the mixed solution of DMF (60 mL), and HCl (10 mL), and 2-aminoterephthalic acid (7 mmol) was dispersed in this mixed solution, followed by ultra-sonication for 20 min under ambient conditions (25 °C). The resulting suspension was transferred into a 100 mL Teflon-lined autoclave and placed in an oven under autogenous pressure at 120 °C for 24 h. After being cooled in the autoclave to room temperature, the resulting material was precipitated by centrifugation (8000 rpm) and washed three times each with DMF and methanol. The as obtained material was soaked overnight in methanol to ensure the complete removal of DMF. Afterward, the product was vacuum dried at 150 °C for 12 h for activation, and finally ground to obtain the UiO-66-NH_2_.

### 2.3. Synthesis of ZIF-8-Loaded UiO-66-NH_2_

ZIF-8-loaded UiO-66-NH_2_ was prepared via the layer-by-layer solution deposition method according to the procedure reported in the previous literature [21]. The amino groups of UiO-66-NH_2_ can act as a covalent linker between seed layers and supports in this reaction [22,23]. The lone pair of electrons on the N-atom of amino groups can form complexation with Zn^2+^ ions, which can chelate with 2-MI to produce ZIF-8 seeded layers in surface of UiO-66-NH_2_. After that, through the layer-by-layer solution deposition method, the continuous and compact ZIF-8 can be formed on the surface of UiO-66-NH_2_. The task consists of the following steps: 0.5 g UiO-66-NH_2_ was dispersed in 0.1 M Zn (NO₃)₂/methanol solution (20 mL) and stirred at 1200 rpm for 2 h under ambient conditions (25 °C), followed by ultrasonic washing with methanol for 20 min and centrifugation to the obtained product. To this, 0.8 M 2-methylimidazole/methanol solution (20 mL) was added and stirred at 1200 rpm for 2 h under ambient condition (25 °C), and followed by ultrasonic washing with methanol for 20 min, and finally vacuum dried at 75 °C for 12 h to get the ZIF-8-loaded UiO-66-NH_2_. The whole process represents one loop and this whole process was repeated for n number of cycles by changing the ZIF-8 content and represented as n-ZIF-8@UiO-66-NH_2_.

### 2.4. Characterization

Crystalline structures of ZIF-8-loaded UiO-66-NH_2_ and UiO-66-NH_2_ were examined by XRD using Bruker D8-ADVANCE (Bruker AXS, Karlsruhe, Germany) with Cu Kα radiation in the angular range, 2θ = 5–50°. FTIR spectrum (KBr pellets as substrate) were recorded on Nicolet 5700 spectrophotometer (Thermo Fisher Scientific, Waltham, MA, USA) in the range from 4000 to 400 cm^−1^. The morphology of the nanoparticles was measured on SEM, (Sirion 200, FEI, Hillsboro, OR, USA) and TEM (Tecnai G2 F20 S-TWIN, FEI, Hillsboro, OR, USA). N_2_ adsorption/desorption isotherms of the synthesized adsorbents was performed at 77 K with Quantachrome AUTOSORB IQ (Quantachrome, FL, USA). Zeta potential analysis of the synthesized adsorbents was performed at 25 °C with Malvern Zetasizer instrument (Nano ZS 90new, Malvern Panalytical, Malvern, UK). UV-Vis absorption spectra were measured on a UV-Vis spectrophotometer (UV-3600, Shimadzu, Kyoto, Japan) in the range from 800 to 200 nm. The pH values were detected by pH meter.

### 2.5. Adsorption Experiments of Dyes

Typical representative dyes with different charges were employed to measure the ability of the adsorbent for selectively adsorbing dyes, for example, MB (664 nm), RhB (554 nm), and MO (464 nm). Typically, 10 mg sample was dispersed into 100 mL dye solution (0–50 mg/L) and stirred continuously for 24 h under ambient condition (25 °C). 0.05 M HCl and NaOH were used to regulate pH of the MB solution. The adsorption capacities were calculated using the following Formula (1):

(1) $$q_{e}=\frac{\left(C_{0}-C_{e}\right)V}{m}$$

where *q_e_* is the adsorption capacity of equilibrium (mg/g) and *C*_0_ and *C_e_* are the initial and final concentrations (mg/L), respectively. *m* is the mass of adsorbent for the adsorption experiments (g), and *V* is the volume of dye solutions (L).

## 3. Results and Discussion

### 3.1. Adsorbents Characterizations

**Figure 1:**
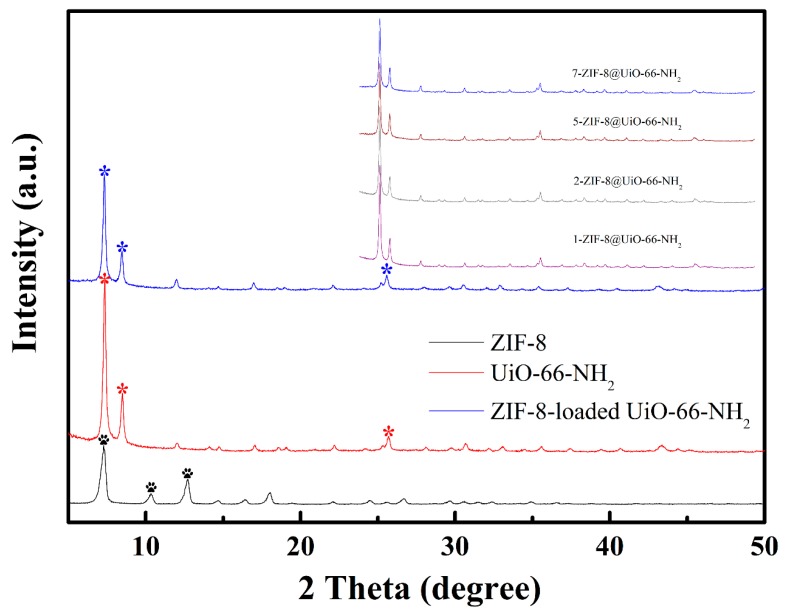
X-ray diffraction (XRD) spectrum of zeolitic imidazolate framework-8 (ZIF-8), UiO-66-NH_2_, ZIF-8-loaded UiO-66-NH_2_. Insets are the XRD spectrum 1-ZIF-8@UiO-66-NH_2_, 2-ZIF-8@UiO-66-NH_2_, 5-ZIF-8@UiO-66-NH_2_, and 7-ZIF-8@UiO-66-NH_2_.

Figure 1 depicts the XRD graphs of the synthesized UiO-66-NH_2_ and ZIF-8-loaded UiO-66-NH_2_. All the characteristic diffraction peaks of UiO-66-NH_2_ and ZIF-8-loaded UiO-66-NH_2_ were sharp, thereby showing excellent crystallinity, which was consistent with the previous reports [21,24]. However, the intensities of diffraction for ZIF-8-loaded UiO-66-NH_2_ were lower than UiO-66-NH_2_, suggesting that the crystallinity of UiO-66-NH_2_ reduced after ZIF-8 loading. It could be due to the interaction between Zn^2+^ and amino groups of ZIF-8 and UiO-66-NH_2_, which might slightly change the frame structure of UiO-66-NH_2_ [25,26]. The diffraction peak of ZIF-8 did not emerge in the XRD pattern of ZIF-8-loaded UiO-66-NH_2_, which is due to the fact that the thickness of ZIF-8 shell was not good enough to form strong scattering crystalline peaks [21].

**Figure 2:**
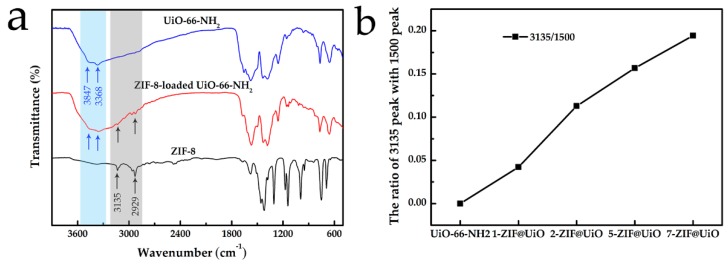
(**a**) Fourier transform infrared spectroscopy (FTIR) spectrum of zeolitic imidazolate framework-8 (ZIF-8)-loaded UiO-66-NH_2_, ZIF-8, and UiO-66-NH_2_. (**b**) semi-quantitative analysis of FTIR spectrum.

The FTIR spectra of ZIF-8-loaded UiO-66-NH_2_ and parent MOFs (UiO-66-NH_2_ and ZIF-8) were analyzed (Figure 2a), aiming to probe the loading amount of ZIF-8. The spectra of UiO-66-NH_2_ exhibited a peak at 1500 cm^−1^ due to C=C of the aromatic group. The peaks at 3368 and 3487 cm^−1^ represent symmetric and asymmetric vibrations of N-H groups, respectively [27]. However, all the characteristic peaks of UiO-66-NH_2_ remained in ZIF-8-loaded UiO-66-NH_2_ and both showed a similar pattern of FTIR spectrum. In addition, two characteristic absorption bands of ZIF-8 at 3135 and 2929 cm^−1^ corresponding to stretching vibration of aromatic and aliphatic C–H, respectively, also appeared, indicating the successful loading of ZIF-8 in the UiO-66-NH_2_ [28]. To determine the loading amount of ZIF-8, semi-quantitative analysis of FTIR with 3135 and 1500 cm^−1^ peaks of ZIF-8 and UiO-66-NH_2_, respectively, was performed (Figure 2b) and found that the ZIF-8 content increased in the UiO-66-NH_2_ on increasing the number of loading cycles.

**Figure 3:**
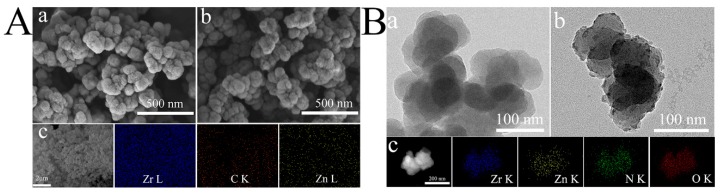
Scanning electron microscopy (SEM) (**A**) and transmission electron microscopy (TEM) (**B**) images of (**a**) UiO-66-NH_2_, (**b**) zeolitic imidazolate framework-8 (ZIF-8)-loaded UiO-66-NH_2_, and (**c**) energy-dispersive X-ray spectroscopy (EDS) mappings of the ZIF-8-loaded UiO-66-NH_2_.

Figure 3A,B displays the images of SEM and TEM for (a) UiO-66-NH_2_, and (b) ZIF-8-loaded UiO-66-NH_2_ (1-ZIF-8@UiO-66-NH_2_), showing that all the samples were agglomerated granules. The shape and size of the UiO-66-NH_2_ and ZIF-8-loaded UiO-66-NH_2_ did not change significantly, and the particle sizes were about 100 nm. Figure 3Ac,Bc displays the EDS mappings of the ZIF-8-loaded UiO-66-NH_2_ and found that the Zn and Zr mappings were consistent with the C, N, and O mappings, indicating that the Zn was uniformly distributed into the UiO-66-NH_2_ framework. TEM was used for a better comparison of difference in morphological structure between UiO-66-NH_2_ and ZIF-8-loaded UiO-66-NH_2_. UiO-66-NH_2_ framework showed smooth surface and no obvious lattice fringes (Figure 3Ba). After loading with ZIF-8, highly dispersed tiny black nanoparticles (about 10–20 nm) were observed on the UiO-66-NH_2_ surface (Figure 3Bb).

**Figure 4:**
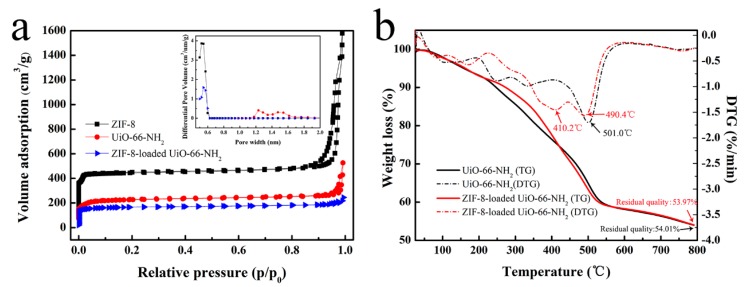
(**a**) N_2_ adsorption/desorption isotherms at 77K (The illustration is pore size distribution). (**b**) Thermogravimetric analysis (TGA) and Derivative Thermogravimetry (DTG) curves for UiO-66-NH_2_ and zeolitic imidazolate framework-8 (ZIF-8)-loaded UiO-66-NH_2_.

N_2_ adsorption/desorption isotherms of UiO-66-NH_2_ and ZIF-8-loaded UiO-66-NH_2_ (1-ZIF-8@UiO-66-NH_2_) are shown in Figure 4a. ZIF-8, UiO-66-NH_2_, and ZIF-8-loaded UiO-66-NH_2_ exhibited strictly Type I isotherm with a predominantly microporous structure. ZIF-8-loaded UiO-66-NH_2_ showed a BET (Brunauer-Emmet-Teller) specific surface area and pore volume of 651 m^2^/g and 0.233 cm^3^/g, respectively, which is much less than ZIF-8 and UiO-66-NH_2_. It might be attributed to the fact that ZIF-8 loaded on the surface of UiO-66-NH_2_ blocked the channels of UiO-66-NH_2_, which further confirmed that ZIF-8 were immobilized on the UiO-66-NH_2_ framework. The parameters of various samples are listed in Table 1. The average pore diameter of as-synthesized UiO-66 was 1.2 nm. However, the pore diameter of UiO-66 decreased to 0.5 nm after loading with ZIF-8. The weight loss of UiO-66-NH_2_ and ZIF-8-loaded UiO-66-NH_2_ (1-ZIF-8@UiO-66-NH_2_) in N_2_ atmosphere is shown in Figure 4b. The first phase of weight loss is attributed to the loss of solvent molecules (H_2_O, DMF) in the temperature range 25–250 °C. The next weight loss is mainly because of destruction of organic framework. Finally, at high temperature, it stabilized, due to the generated metallic oxides. The trend of curves of ZIF-8-loaded UiO-66-NH_2_ were similar to that of UiO-66-NH_2_, which shows that the thermal stability of UiO-66-NH_2_ did not change after loading with ZIF-8.

### 3.2. Separation Selectivity in the Mixed Dye Solution

The ideal adsorption selectivity of *MB* over *MO* and *RhB* on ZIF-8-loaded UiO-66-NH_2_ was calculated according to the following expression:

(2) S=qMBCMBqMO(RhB)CMO(RhB)

where *S* is the dye selective adsorption index, *q_MB_*, *q_MO_*, and *q_RhB_* are the adsorption capacity of dye (*MB*, *MO*, and *RhB*, respectively) on adsorbents when in equilibrium (mg/g), *C_MB_*, *C_MO_*, and *C_RhB_* correspond to initial concentration of different dyes in mixed solution (mg/L).

**Figure 5:**
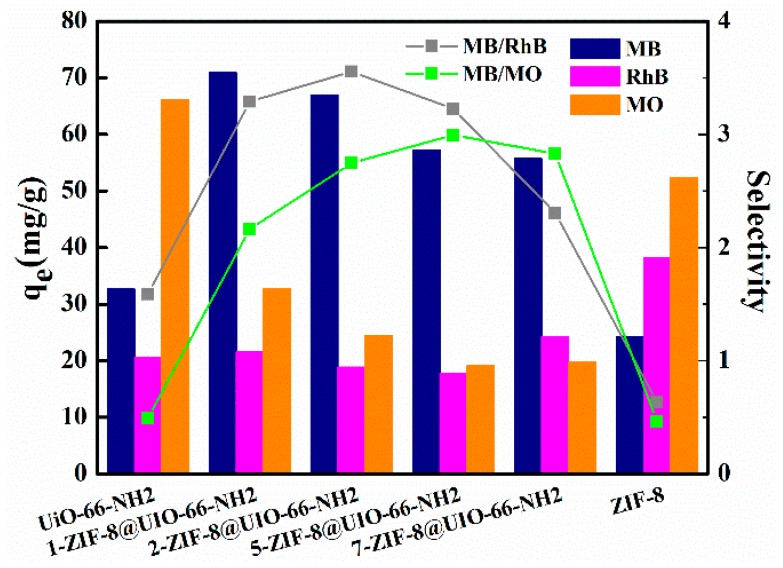
The selective adsorption of different adsorbents in mixed dyes.

As shown in Figure 5, the adsorptive capacity of ZIF-8-loaded UiO-66-NH_2_ for MB was significantly higher than its parent MOFs (UiO-66-NH_2_ and ZIF-8). At the same time, the adsorption selectivity of MB over MO or RhB first increased and then decreased with increasing ZIF-8 loading. Pure UiO-66-NH_2_ exhibited the best adsorption effect on MO in mixed dyes due to the addition of HCl as a regulator during preparation. The dye preferential sorption was due to the hydrogen ions exposure on to the acid-promoted UiO-66-NH_2_ surface and resulted in zeta potential raise [29]. Pure ZIF-8 also showed the best adsorption effect on MO in mixed dyes due to the positive charge of its surface [30]. However, ZIF-8-loaded UiO-66-NH_2_ exhibited the best adsorption effect on MB in mixed dyes. Among them, 1-ZIF-8@UiO-66-NH_2_ showed the largest adsorptive capacity of MB. Owing to the opposite electrical properties of MB and MO, the zeta potential of ZIF-8-loaded UiO-66-NH_2_ could be lower than UiO-66-NH_2_. For the better insight of adsorption interactions between ZIF-8-loaded UiO-66-NH_2_ and MB, the adsorption of MB by 1-ZIF-8@UiO-66-NH_2_ has been discussed in detail in the proceeding sections.

### 3.3. Adsorption Isotherms

To understand the adsorption mechanisms of *MB* with ZIF-8-loaded UiO-66-NH_2_, Langmuir (3) and Freundlich (4) isotherm models were studied.

(3) $$\frac{C_{e}}{q_{e}}=\frac{1}{K_{L}q_{max}}+\frac{C_{e}}{q_{max}}$$

(4) $$lnq_{e}=lnK_{F}+\frac{1}{n}lnC_{e}$$

where, *q_max_* is maximum adsorption capacity (mg/g), *K_F_* and *K_L_* are Freundlich constant and Langmuir constant, respectively, and 1/*n* is Freundlich constant related to the adsorption.

**Figure 6:**
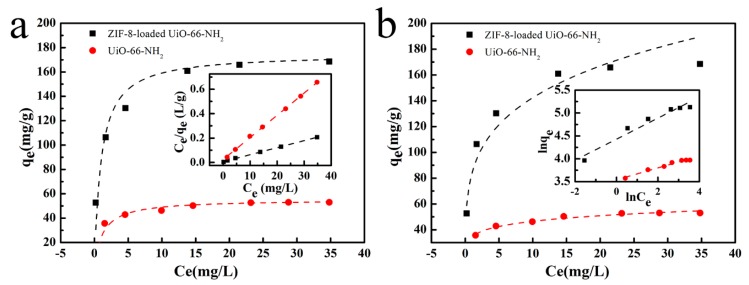
Langmuir (**a**) and Freundlich (**b**) isotherm models for the adsorption of methylene blue (MB) on UiO-66-NH_2_ and zeolitic imidazolate framework-8 (ZIF-8)-loaded UiO-66-NH_2_.

Figure 6 shows that the adsorption of *MB* by the UiO-66-NH_2_ and ZIF-8-loaded UiO-66-NH_2_ conforms to Langmuir isotherm model, while the predicted result of Freundlich adsorption model was significantly different from experimental data for *MB*. The model parameters for adsorption are presented in the Table 2. Correlation coefficient (R^2^) of Langmuir isotherm models were bigger than that of Freundlich isotherm models and R^2^ value of Langmuir isotherm models was almost equal to unity, which suggested that the adsorption of *MB* on these two adsorbents was heterogeneous and a monolayer adsorption process [31]. The theoretical maximum adsorption capacity of the ZIF-8-loaded UiO-66-NH_2_ (173 mg/g) was higher than pure UiO-66-NH_2_ (55 mg/g), indicating 215% improvement in the adsorption capacity, which could be due to the loading of ZIF-8 nanoparticles. To date, several researches have been carried out for the removal of *MB* by *MOFs* [32]. Table 3 compares the adsorption capacities of *MB* over similar MOFs under the same experimental conditions [29,33,34,35,36,37]. Yang [36] demonstrated the use of UiO-66-P for the adsorptive removal of *MB*, *MO*, Congo Red (CR), and Acid Chrome Blue K (AC). UiO-66-P could adsorb *MB* but not the other dyes. The adsorption capability of UiO-66-P (91.1 mg/g) for *MB* was improved by 272% compared to that of pristine UiO-66 (24.5 mg/g), which shows that the negative charge of UiO-66 was due to phosphate immobilization approach. Yang et al. [37] studied the adsorption of *MB* using Ce(III)-doped UiO-66 and showed decrease in surface potential of UiO-66, thereby enhancing adsorption capacity for *MB*. However, the surface potential of UiO-66-P (−12.6 mv) was lower than Ce(III)-doped UiO-66 (+6.48 mv) but the adsorption capacity of MB on Ce(III)-doped UiO-66 (145.1 mg/g) higher than UiO-66-P. This behavior could be due to higher specific surface area of synthesized UiO-66 composite, which indicates that the adsorption process was controlled by many factors. These results further confirmed that ZIF-8-loaded UiO-66-NH_2_ demonstrates enormous advantage for MB in adsorption capacity and selective adsorption.

### 3.4. Kinetic Studies

In order to better explore the adsorption rate, kinetic models were studied. The kinetic equation shows the relationship between concentration and other parameters with time. The kinetics models of pseudo-first-order (5) and pseudo-second-order (6) are as follows [38,39,40]:

(5) $$\mathrm{lg}\left(q_{e}-q_{t}\right)=lgq_{e}-K_{1}\frac{t}{2.303}$$

(6) $$\frac{t}{q_{t}}=\frac{1}{K_{2}q_{e}^{2}}+\frac{t}{q_{e}}$$

where *K*_1_ and *K*_2_ are the rate constant of pseudo-first-order kinetics model (min^−1^) and pseudo-second-order kinetics model (g/mg min), respectively. t is the adsorption time (min). The values of the kinetic parameters are set out in the Table 4.

**Figure 7:**
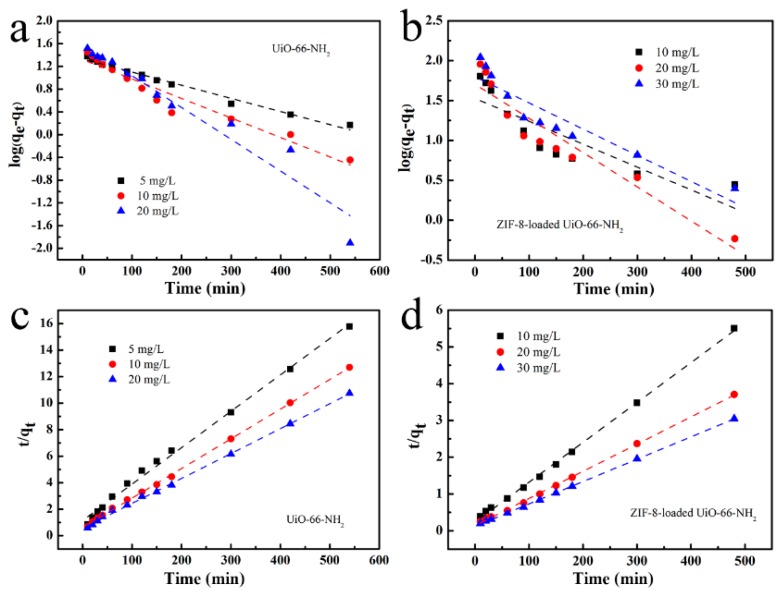
Pseudo-first-order and pseudo-second-order kinetics model for adsorption of methylene blue (MB) onto UiO-66-NH_2_ (**a**,**c**) and zeolitic imidazolate framework-8 (ZIF-8)-loaded UiO-66-NH_2_ (**b**,**d**).

Figure 7 shows the pseudo-first-order and pseudo-second-order kinetic models for the adsorption of *MB* onto UiO-66-NH_2_ and ZIF-8-loaded UiO-66-NH_2_, and displays that the latter fitted with better linearity compared to former. Moreover, the calculated data of the kinetics model for pseudo-second-order was very close to the adsorption data, which showed that the process of adsorption in our work conforms to the kinetics model of pseudo-second-order.

Liquid phase adsorption generally consists of three parts [41]: Step one represents film transport involving the diffusion of pollutants through a boundary layer of hypothetical film for the adsorbent. The second step represents intra-particle diffusion, and the last step represents adsorption and desorption to achieve balance. Film and intra-particle transport are the main constraints that control the absorption rates by porous adsorbents. In our experiments, using the model of intra-particle diffusion, we simulated the adsorption process in order to understand the diffusion mechanism between the adsorbent and MB [42]. The related model is as following (7):

(7) $$q_{t}=k_{i}^{\frac{1}{2}}+C_{i}$$

where *k_i_* is the rate constant of intra-particle diffusion during *i* stage (mg/g min)^1/2^ and *C_i_* is constant of boundary layer theory. The parameters of the model are listed in Table 5.

**Figure 8:**
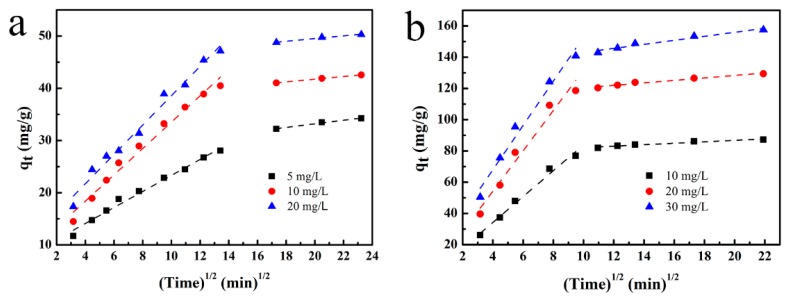
Intra-particle diffusion kinetic model for the adsorption of methylene blue (MB) on UiO-66-NH_2_ (**a**) and zeolitic imidazolate framework-8 (ZIF-8)-loaded UiO-66-NH_2_ (**b**).

Figure 8 shows that the curves were divided into two regions, which suggested that the adsorption process contains two or more steps. The first half of the curve represents the adsorbed molecules diffusion in the fluid film on the surface of the particles. The slope of the second part of the curve was significantly smaller, indicating the lower adsorption rate of this part. This behavior represents the process of intra-particle diffusion. Using a model of intra-particle diffusion, all R^2^ values were > 0.9, which indicates that adsorption of MB on UiO-66-NH_2_ and ZIF-8-loaded UiO-66-NH_2_ could be described by intra-particle diffusion. Therefore, the adsorption of MB by the ZIF-8-loaded UiO-66-NH_2_ or UiO-66-NH_2_ was based on both the diffusion of external and intra-particle. Moreover, the coordinate origin was not passed by the curve, which signified that the diffusion of intra-particle was not the only dominant factor of adsorption rate [43].

### 3.5. Effect of pH on Adsorption

**Figure 9:**
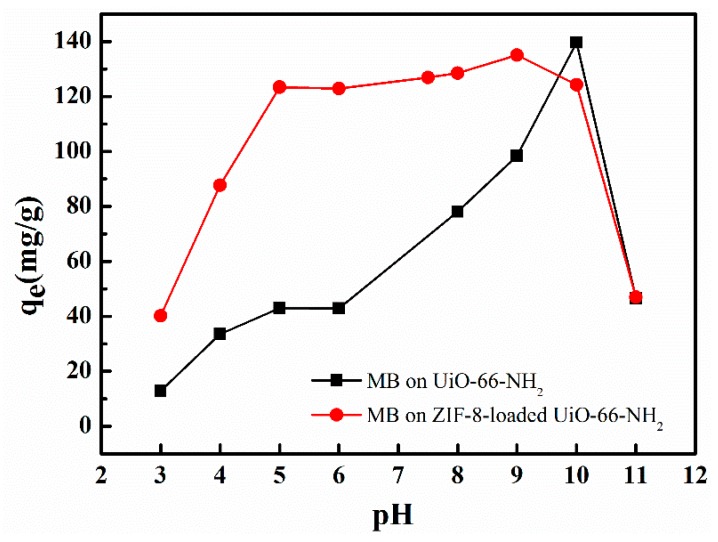
The effect of pH on methylene blue (MB) adsorption onto UiO-66-NH_2_ and zeolitic imidazolate (ZIF-8)-loaded UiO-66-NH_2_ at ambient condition (25 °C).

The pH of the dye solution has a huge influence on the dye adsorption capacity of absorbent. Figure 9 represents the impact of various pH on MB adsorption onto UiO-66-NH_2_ and ZIF-8-loaded UiO-66-NH_2_ under ambient conditions (25 °C). Due to the fact that MB aqueous solution can result in the blue shift in absorption wavelength under strongly alkaline conditions, the adsorption experiments were carried out only under acidic and weakly basic conditions (pH 3–11). For UiO-66-NH_2_, the adsorptive capacity of MB increased rapidly on raising the pH to 10. Nevertheless, the adsorption capacity decreased with pH >10. At low pH, the UiO-66-NH_2_ with positive surface charge hinders the adsorption of cationic dye, MB. The highest adsorptive capacity was observed at pH = 10, which indicates that the hydroxyl ion promoted the adsorption of MB. However, the adsorption capacity of MB was lowered with pH > 10, which may be due to excessive hydroxide ions that might compete with the adsorbent, leading to the decrease in adsorption capacity [44]. For ZIF-8-loaded UiO-66-NH_2_, the adsorption capacity of MB increased rapidly as the pH rose to 5 and the adsorption capacity of MB remained stable at pH = 5–10. Therefore, it can be concluded that the stability of MB on ZIF-8-loaded UiO-66-NH_2_ was higher compared with UiO-66-NH_2_. From 5 to 3, the adsorption capacity of the ZIF-8-loaded UiO-66-NH_2_ rapidly decreased, which may be due to the fact that ZIF-8 might dissolve in acidic conditions [45].

### 3.6. Adsorption Thermodynamics

To understand the adsorption mechanism of MB with ZIF-8-loaded UiO-66-NH_2_, the change in adsorption capacity of MB onto UiO-66-NH_2_ and ZIF-8-loaded UiO-66-NH_2_ under different temperatures (25, 35, and 45 °C) was studied. In general, the parameters were calculated by the following Equations [46]:

(8) $$\ln\frac{q_{e}}{c_{e}}=\frac{\Delta S^{0}}{R}-\frac{\Delta H^{0}}{RT}$$

(9) $$\Delta G^{0}=\Delta H^{0}-T\Delta S^{0}.\;$$

where Δ*S°*, Δ*H°*, and Δ*G°* are the standard entropy change (J/mol K), enthalpy changes (kJ/mol), and free energy (kJ/mol), respectively. *T* and *R* are the adsorption temperature (K) and gas constant (8.3145 J/mol K), respectively. Table 6 shows the thermodynamic parameter values.

**Figure 10:**
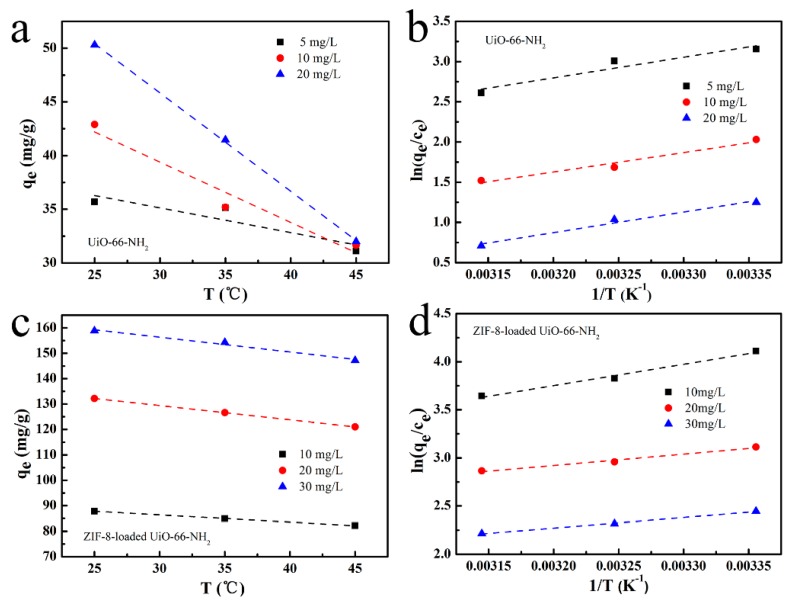
Effect of temperature on the adsorption capacity of methylene blue (MB) on UiO-66-NH_2_ (**a**) and zeolitic imidazolate framework-8 (ZIF-8)-loaded UiO-66-NH_2_ (**c**). Plots of ln(q_e_/c_e_) versus 1/T for the adsorption of MB on UiO-66-NH_2_ (**b**) and ZIF-8-loaded UiO-66-NH_2_ (**d**).

The negative values of enthalpy change show that the adsorption processes were exothermic, which was in accordance with the results in Figure 10, in which the adsorption capacity of MB decreased with the rise of temperature. Moreover, the value of adsorption heat on UiO-66-NH_2_ was about −20 kJ/mol, which shows that the interaction between UiO-66-NH_2_ and MB could be because of the dipole-dipole interaction and/or hydrogen-bond [47]. By comparison, the value of adsorption heat for ZIF-8-loaded UiO-66-NH_2_ slightly declined, which suggested that it may have other interaction. Based on the correlative references, the ΔG° of chemisorption and physisorption were −400 to −50 kJ/mol and −40 to 0 kJ/mol, respectively [48]. The free energy in our case was found to be negative, which indicates that the adsorption course was an irreversible and spontaneous processes. The values of free energy of UiO-66-NH_2_ and ZIF-8-loaded UiO-66-NH_2_ were in range of −11 to −5 (kJ/mol) and −8 to −1 (kJ/mol), respectively, which suggests that both the adsorption course of MB were primarily physisorption. Furthermore, the negative value of entropy change shows that MB mobility decreased after adsorption with UiO-66-NH_2_ or ZIF-8-loaded UiO-66-NH_2_. Since both entropy change and enthalpy change were negative, it can be concluded that the enthalpy change is an important driving force with such kind of adsorption processes.

### 3.7. Reusability of ZIF-8-Loaded UiO-66-NH_2_

**Figure 11:**
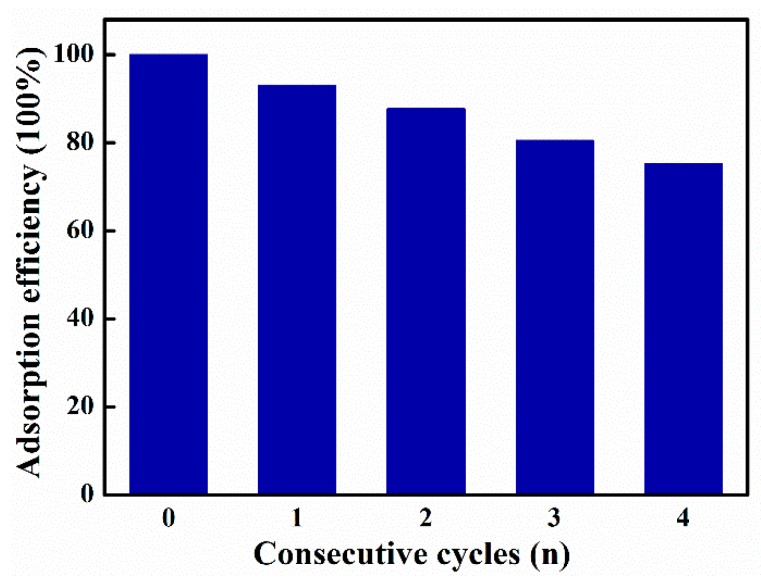
Recycling performance of regenerated zeolitic imidazolate framework-8 (ZIF-8)-loaded UiO-66-NH_2_.

The recycling adsorption experiments were performed on 1-ZIF-8@UiO-66-NH_2_ in order to analyze the reusability of ZIF-8-loaded UiO-66-NH_2_. The used adsorbent recovered by centrifugation was immersed into 100 mL ethanol for desorption by ultrasonication for 60 min and washed with methanol several times, followed by drying at 373 K for the next adsorption cycle. The recycling performance of ZIF-8-loaded UiO-66-NH_2_ (Figure 11) indicates the significant regeneration efficiency in adsorption capacity for MB after four adsorption cycles (less than 25% decrease in adsorption capacity), which provides its applicability to regenerate and reuse in actual dye removal.

### 3.8. Adsorption Mechanism

**Figure 12:**
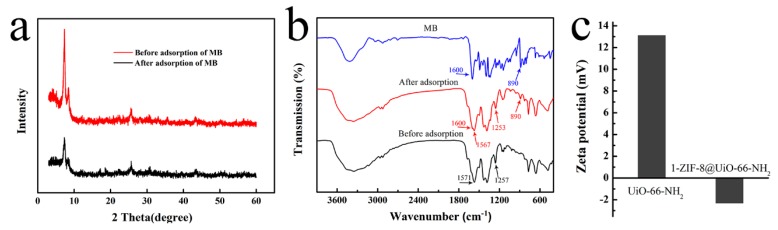
(**a**) X-ray diffraction (XRD) and (**b**) Fourier transform infrared spectroscopy (FT-IR) of zeolitic imidazolate framework-8 (ZIF-8)-loaded UiO-66-NH_2_ before adsorption and recovered after adsorption of methylene blue (MB), and (**c**) zeta potentials of UiO-66-NH_2_ and ZIF-8-loaded UiO-66-NH_2_.

**Scheme 1:**
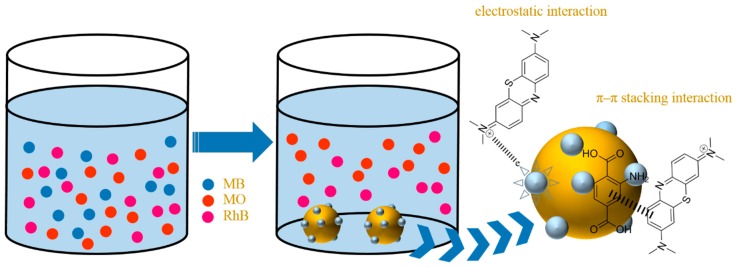
The possible adsorption mechanism of dyes adsorption on zeolitic imidazolate framework-8 (ZIF-8)-loaded UiO-66-NH_2._

To further reveal the MB adsorption mechanism, XRD and FT-IR analysis of ZIF-8-loaded UiO-66-NH_2_ before and after adsorption of MB were examined. XRD spectra (Figure 12a) showed all the peaks of ZIF-8-loaded UiO-66-NH_2_ at the same position after the adsorption of MB, demonstrating the unchanged crystalline structure of ZIF-8-loaded UiO-66-NH_2_. Nevertheless, the characteristic peak intensities of ZIF-8-loaded UiO-66-NH_2_ recovered after adsorption was found to be weaker than ZIF-8-loaded UiO-66-NH_2_ before used, which may be due to binding of dye molecules with the active binding sites of adsorbents [49]. In the FTIR spectra, after adsorption of MB on ZIF-8-loaded UiO-66-NH_2_ (Figure 12b), an intense and broad band centered at 3368 cm^−1^ remain unchanged (the broad band could be due to peak overlap of N–H and O–H [50]), which showed that the hydrogen bonding might not play a major role in the adsorption process [51]. However, the new absorption peaks for the vibration of aromatic ring of MB at 890 cm^−1^ and 1600 cm^−1^ could be clearly observed [52], which showed that the MB was immobilized on surface of ZIF-8-loaded UiO-66-NH_2_. The peaks at 1571 cm^−1^ (C=O), 1257 cm^−1^ (C–N), and 426 cm^−1^ (Zn–N) of ZIF-8-loaded UiO-66-NH_2_ shifted and showed a significant decrease in intensity after MB adsorption [28,53,54]. This could be explained by the following assumptions: (i) MB, a cationic dye, is more inclined to be absorbed on negatively charged surfaces by electrostatic interaction. The surface potential of the UiO-66-NH_2_ changed from positive (+13) to negative (−2) after ZIF-8 loading as revealed by zeta potential (Figure 12c) and the cause of the change in surface potential might be because the basic sites (OH^−^ groups and N^−^ moieties mainly) adsorbed on the surface of ZIF-8 in the solution [55], which could provide the adsorption sites for electrostatic interaction with MB. (ii) Both MB and ZIF-8-loaded UiO-66-NH_2_ contain aromatic rings and π–π stacking interactions could occur between MB and ZIF-8-loaded UiO-66-NH_2_, resulting in the shift of the peak (1257 to 1253 cm^−1^; 1571 to 1567 cm^−1^) [56]. Moreover, the characteristics of good water stability and relatively high specific surface area make ZIF-8-loaded UiO-66-NH_2_ contact and adsorb MB efficiently, which provides an advantage for the ZIF-8-loaded UiO-66-NH_2_ to adsorb the cationic dyes. However, RhB is also cationic dye, but the bad performance of ZIF-8-loaded UiO-66-NH_2_ in adsorption of RhB might be due to the steric hindrance because of large molecular size. The complex structure of RhB (as shown in Table 7) with bigger molecular size compared with MB might hinder it from entering the pores of ZIF-8-loaded UiO-66-NH_2_ (RhB: 1.41 × 0.98 nm > pore width: 0.5 nm). In conclusion, the ZIF-8-loaded UiO-66-NH_2_ was suitable for selective adsorption of small-size cationic dye and their interaction was mainly affected by electrostatic and π–π stacking interactions. The possible adsorption mechanism is schematically illustrated in Scheme 1.

## 4. Conclusions

In summary, the positively charged UiO-66-NH_2_ was synthesized successfully using the solvothermal method and ZIF-8 was successfully loaded into UiO-66-NH_2_, as revealed by FTIR and XRD. The content of ZIF-8 present after several loading cycles was determined by semi-quantitative analysis of FTIR spectrum. The adsorption capacity of ZIF-8-loaded UiO-66-NH_2_ (173 mg/g) toward MB was found to be 215% higher than UiO-66-NH_2_ (55 mg/g), which was due to the improved electrostatic attraction between MB and UiO-66-NH_2_ after loading with ZIF-8. Kinetic studies indicated that the adsorption process in this work conforms to the pseudo-second-order kinetics model and the adsorption of MB by the ZIF-8-loaded UiO-66-NH_2_ or UiO-66-NH_2_ was based on the joint control of the diffusion of external and intra-particle. The adsorption capacity of MB by ZIF-8-loaded UiO-66-NH_2_ increased rapidly as the pH rose to 5 and remained stable at pH = 5–10. The recycling adsorption experiments revealed the significant regeneration efficiency in adsorption capacity for MB even after four adsorption cycles and displayed no change in the crystalline structure of the reused ZIF-8-loaded UiO-66-NH_2_ as revealed by XRD and IR. Thus, the prepared ZIF-8-loaded UiO-66-NH_2_ showed that high selectivity and good water stability can be of great potential in the water treatment.

## Figures and Tables

**Table 1:** Pore structure parameters of zeolitic imidazolate framework-8 (ZIF-8), UiO-66-NH_2_, 1-ZIF-8@UiO-66-NH_2._

Sample	S_BET_ (m^2^/g)	S_micro_ (m^2^/g)	V_micro_ (cm^3^/g)	Pore Width (nm)
ZIF-8	1938.859	1841.897	0.646	0.524
UiO-66-NH_2_	897.779	801.012	0.308	1.232
1-ZIF-8@UiO-66-NH_2_	651.895	596.664	0.233	0.548

**Table 2:** Values of isotherm model parameters for the adsorption of methylene blue (MB) on UiO-66-NH_2_ and zeolitic imidazolate framework-8 (ZIF-8)-loaded UiO-66-NH_2._

Adsorbent	Langmuir Isotherm				Freundlich Isotherm			
	q_max_	K_L_	R_L_	R^2^	K_F_	1/n	n	R^2^
1-ZIF@UiO	173.01	1	0.0175	0.999	83.89	0.2297	4.35	0.937
UiO-66-NH_2_	55.04	0.79	0.0324	0.999	34.50	0.1297	7.71	0.972

**Table 3:** Comparison of methylene blue (MB) maximum adsorption capacities on various metal organic frameworks (MOFs).

Adsorbents	q_max_ (mg/g)	References
UiO-66	69.8	[33]
UiO-66	90	[34]
UiO-66-NH_2_	90.88	[35]
Acid- promoted UiO-66	13.0	[29]
UiO-66-P composite	91.1	[36]
Ce(III)-doped UiO-66	145.1	[37]
UiO-66-NH_2_	55	Our work
ZIF-8-loaded UiO-66-NH_2_	173	Our work

**Table 4:** Values of the kinetic parameters for 1-zeolitic imidazolate framework-8 (ZIF-8)@UiO-66-NH_2_ and UiO-66-NH_2_.

Adsorbent	C_0_	q_e_	Pseudo-First-Order Kinetic			Pseudo-Second-Order Kinetic			
			q_e_	K_1_	R^2^	q_e_	K_2_	R^2^	Rate
1-ZIF@UiO	10	90	33.64	0.0066	0.727	92.25	0.00050	0.999	0.046
	20	132	50.68	0.0099	0.897	135.32	0.00039	0.999	0.053
	30	160	62.90	0.0076	0.866	164.47	0.00030	0.999	0.049
UiO-66-NH_2_	5	36	21.48	0.0053	0.984	36.44	0.00068	0.995	0.025
	10	43	21.03	0.0079	0.937	44.74	0.00083	0.999	0.037
	20	50	39.23	0.0129	0.941	53.22	0.00062	0.999	0.033

**Table 5:** Parameters of intra-particle diffusion kinetic model.

Adsorbent	C (mg/L)	Intra-Particle Diffusion Kinetic Model					
		K_1_	C_1_	R^2^	K_2_	C_2_	R^2^
1-ZIF@UiO	10	8.27	1.16	0.983	0.47	77.48	0.913
	20	12.91	2.54	0.959	0.79	112.42	0.961
	30	14.17	11.27	0.972	1.28	130.13	0.950
UiO-66-NH_2_	5	1.53	7.97	0.985	0.34	26.40	0.982
	10	2.52	8.29	0.981	0.26	36.58	0.995
	20	2.81	10.48	0.982	0.26	44.31	0.964

**Table 6:** Values of the thermodynamic parameters.

Adsorbent	CONCENTRATION (mg/L)	ΔH° (kJ/mol)	ΔS° (J/mol K)	ΔG° (kJ/mol)			
				298 K	308 K	318 K	R^2^
1−ZIF@UiO	10	−18.46	−27.89	−10.15	−9.87	−9.59	0.977
	20	−9.83	−7.18	−7.69	−7.62	−7.55	0.970
	30	−9.11	−10.29	−6.05	−5.95	−5.84	0.996
UiO−66−NH_2_	5	−21.31	−44.93	−7.92	−7.47	−7.02	0.850
	10	−20.11	−50.84	−4.96	−4.46	−3.95	0.936
	20	−21.29	−60.86	−3.15	−2.54	−1.93	0.963

**Table 7:** Structure of dyes.

Adsorbate	Molecular Structure	Size (nm)
Methylene blue (MB^+^)	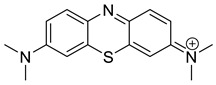	1.38 × 0.64
Methyl orange (MO^−^)	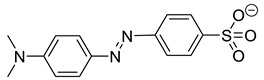	1.54 × 0.48
Rhodamine B (RhB^+^)	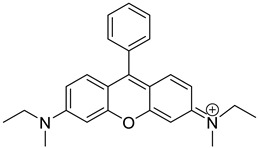	1.41 × 0.98

## References

[1] Zhou H.-C., Long J.R., Yaghi O.M. (2012). Introduction to Metal–Organic Frameworks. Chem. Rev..

[2] Dou Y., Rutledge W., Bai Y., Xie L.-H., Li J.-R., Zhou H.-C. (2016). Zr-based metal–organic frameworks: Design, synthesis, structure, and applications. Chem. Soc. Rev..

[3] Zeng L., Xiao L., Long Y., Shi X. (2018). Trichloroacetic acid-modulated synthesis of polyoxometalate@UiO-66 for selective adsorption of cationic dyes. J. Colloid Interface Sci..

[4] He J., Wang J., Chen Y., Zhang J., Duan D., Wang Y., Yan Z. (2014). A dye-sensitized Pt@UiO-66(Zr) metal–organic framework for visible-light photocatalytic hydrogen production. Chem. Commun..

[5] Gong Y., Yuan Y., Chen C., Zhang P., Wang J., Khan A., Zhuiykov S., Chaemchuen S., Verpoort F. (2019). Enhancing catalytic performance via structure core-shell metal-organic frameworks. J. Catal..

[6] Koh K., Wong-Foy A.G., Matzger A.J. (2009). MOF@MOF: Microporous core-shell architectures. Chem. Commun..

[7] Ren J., Musyoka N.M., Langmi H.W., North B.C., Mathe M., Kang X. (2014). Fabrication of core–shell MIL-101(Cr)@UiO-66(Zr) nanocrystals for hydrogen storage. Int. J. Hydrog. Energy.

[8] Zhuang J., Chou L.-Y., Sneed B.T., Cao Y., Hu P., Feng L., Tsung C.-K. (2015). Surfactant-Mediated Conformal Overgrowth of Core-Shell Metal-Organic Framework Materials with Mismatched Topologies. Small.

[9] Tambat S.N., Sane P.K., Suresh S., O. N.V., Pandit A.B., Sontakke S.M., Varadan O.N. (2018). Hydrothermal synthesis of NH_2_-UiO-66 and its application for adsorptive removal of dye. Adv. Powder Technol..

[10] Fan J., Chen D., Li N., Xu Q., Li H., He J., Lu J. (2018). Adsorption and biodegradation of dye in wastewater with Fe_3_O_4_@MIL-100 (Fe) core–shell bio-nanocomposites. Chemosphere.

[11] Li H., Li Q., He X., Xu Z., Wang Y., Jia L. (2019). Synthesis of AgBr@MOFs nanocomposite and its photocatalytic activity for dye degradation. Polyhedron.

[12] Wang K., Qin Y., Quan S., Zhang Y., Wang P., Liang H., Ma J., Cheng X.Q. (2019). Development of highly permeable polyelectrolytes (PEs)/UiO-66 nanofiltration membranes for dye removal. Chem. Eng. Res. Des..

[13] Mittal H., Maity A., Sinha Ray S. (2015). The adsorption of Pb^2+^ and Cu^2+^ onto gum ghatti-grafted poly(acrylamide-co-acrylonitrile) biodegradable hydrogel: Isotherms and kinetic models. J. Phys. Chem. B.

[14] Kyo S.P., Zheng N., Adrien P.C., Jae Y.C., Rudan H., Fernando J.U., Hee K.C., Michael O., Omar M.Y. (2006). Exceptional chemical and thermal stability of zeolitic imidazolate frameworks. Proc. Natl. Acad. Sci. USA.

[15] Li Y., Zhou K., He M., Yao J. (2016). Synthesis of ZIF-8 and ZIF-67 using mixed-base and their dye adsorption. Microporous Mesoporous Mater..

[16] Yoon S., Calvo J.J., So M.C. (2018). Removal of Acid Orange 7 from Aqueous Solution by Metal-Organic Frameworks. Crystals.

[17] Feng Y., Li Y., Xu M., Liu S., Yao J. (2016). Fast adsorption of methyl blue on zeolitic imidazolate framework-8 and its adsorption mechanism. RSC Adv..

[18] Thanh M.T., Thien T.V., Chau V.T.T., Du P.D., Hung N.P., Khieu D.Q. (2017). Synthesis of Iron Doped Zeolite Imidazolate Framework-8 and Its Remazol Deep Black RGB Dye Adsorption Ability. J. Chem..

[19] Zhou L., Li N., Owens G., Chen Z. (2019). Simultaneous removal of mixed contaminants, copper and norfloxacin, from aqueous solution by ZIF-8. Chem. Eng. J..

[20] Katz M.J., Brown Z.J., Colón Y.J., Siu P.W., Scheidt K.A., Snurr R.Q., Hupp J.T., Farha O.K. (2013). A facile synthesis of UiO-66, UiO-67 and their derivatives. Chem. Commun..

[21] Song Z., Qiu F., Zaia E.W., Wang Z., Kunz M., Guo J., Brady M., Mi B., Urban J.J. (2017). Dual-Channel, Molecular-Sieving Core/Shell ZIF@MOF Architectures as Engineered Fillers in Hybrid Membranes for Highly Selective CO_2_ Separation. Nano Lett..

[22] Huang A., Bux H., Steinbach F., Caro J. (2010). Molecular-Sieve Membrane with Hydrogen Permselectivity: ZIF-22 in LTA Topology Prepared with 3-Aminopropyltriethoxysilane as Covalent Linker. Angew. Chem. Int. Ed..

[23] Huang K., Dong Z., Li Q., Jin W. (2013). Growth of a ZIF-8 membrane on the inner-surface of a ceramic hollow fiber via cycling precursors. Chem. Commun..

[24] Yuan G., Tu H., Liu J., Zhao C., Liao J., Yang Y., Yang J., Liu N. (2018). A novel ion-imprinted polymer induced by the glycylglycine modified metal-organic framework for the selective removal of Co(II) from aqueous solutions. Chem. Eng. J..

[25] Xu G., Yao J., Wang K., He L., Webley P.A., Chen C.-S., Wang H. (2011). Preparation of ZIF-8 membranes supported on ceramic hollow fibers from a concentrated synthesis gel. J. Membr. Sci..

[26] Hwang Y.K., Hong D.-Y., Chang J.-S., Jhung S.H., Seo Y.-K., Kim J., Vimont A., Daturi M., Serre C., Férey G. (2008). Amine Grafting on Coordinatively Unsaturated Metal Centers of MOFs: Consequences for Catalysis and Metal Encapsulation. Angew. Chem. Int. Ed..

[27] Kandiah M., Usseglio S., Svelle S., Olsbye U., Lillerud K.P., Tilset M. (2010). Post-synthetic modification of the metal–organic framework compound UiO-66. J. Mater. Chem. C.

[28] Beh J.J., Lim J.K., Ng E.P., Ooi B.S. (2018). Synthesis and size control of zeolitic imidazolate framework-8 (ZIF-8): From the perspective of reaction kinetics and thermodynamics of nucleation. Mater. Chem. Phys..

[29] Qiu J., Feng Y., Zhang X., Jia M., Yao J. (2017). Acid-promoted synthesis of UiO-66 for highly selective adsorption of anionic dyes: Adsorption performance and mechanisms. J. Colloid Interface Sci..

[30] Li H., Tuo L., Yang K., Jeong H.-K., Dai Y., He G., Zhao W. (2016). Simultaneous enhancement of mechanical properties and CO_2_ selectivity of ZIF-8 mixed matrix membranes: Interfacial toughening effect of ionic liquid. J. Membr. Sci..

[31] Zhu B.-J., Yu X.-Y., Jia Y., Peng F.-M., Sun B., Zhang M.-Y., Luo T., Liu J.-H., Huang X.-J. (2012). Iron and 1,3,5-Benzenetricarboxylic Metal–Organic Coordination Polymers Prepared by Solvothermal Method and Their Application in Efficient As(V) Removal from Aqueous Solutions. J. Phys. Chem. C.

[32] Dias E.M., Petit C. (2015). Towards the use of metal–organic frameworks for water reuse: A review of the recent advances in the field of organic pollutants removal and degradation and the next steps in the field. J. Mater. Chem. A.

[33] Molavi H., Hakimian A., Shojaei A., Raeiszadeh M. (2018). Selective dye adsorption by highly water stable metal-organic framework: Long term stability analysis in aqueous media. Appl. Surf. Sci..

[34] Mohammadi A.A., Alinejad A., Kamarehie B., Javan S., Ghaderpoury A., Ahmadpour M., Ghaderpoori M. (2017). Metal-organic framework Uio-66 for adsorption of methylene blue dye from aqueous solutions. Int. J. Environ. Sci. Technol..

[35] Chen Q., He Q., Lv M., Xu Y., Yang H., Liu X., Wei F. (2015). Selective adsorption of cationic dyes by UiO-66-NH_2_. Appl. Surf. Sci..

[36] Yang J.-M. (2017). A facile approach to fabricate an immobilized-phosphate zirconium-based metal-organic framework composite (UiO-66-P) and its activity in the adsorption and separation of organic dyes. J. Colloid Interface Sci..

[37] Yang J.-M., Ying R.-J., Han C.-X., Hu Q.-T., Xu H.-M., Li J.-H., Wang Q., Zhang W. (2018). Adsorptive removal of organic dyes from aqueous solution by a Zr-based metal-organic framework: Effects of Ce(iii) doping. Dalton Trans..

[38] Mahdavi H., Ahmadian-Alam L., Molavi H., Ahmadian-Alam L. (2015). Grafting of sulfonated monomer onto an amino-silane functionalized 2-aminoterephthalate metal—Organic framework via surface-initiated redox polymerization: Proton-conducting solid electrolytes. Polym. Int..

[39] Aghajanzadeh M., Zamani M., Molavi H., Manjili H.K., Danafar H., Shojaei A. (2017). Preparation of Metal–Organic Frameworks UiO-66 for Adsorptive Removal of Methotrexate from Aqueous Solution. J. Inorg. Organomet. Polym. Mater..

[40] Molavi H., Zamani M., Aghajanzadeh M., Manjili H.K., Danafar H., Shojaei A. (2018). Evaluation of UiO-66 metal organic framework as an effective sorbent for Curcumin’s overdose. Appl. Organomet. Chem..

[41] Fisal A., Daud W.M.A.W., Ahmad M.A., Radzi R. (2011). Using cocoa (Theobroma cacao) shell-based activated carbon to remove 4-nitrophenol from aqueous solution: Kinetics and equilibrium studies. Chem. Eng. J..

[42] Darwish A., Rashad M., Al-Aoh H.A. (2019). Methyl orange adsorption comparison on nanoparticles: Isotherm, kinetics, and thermodynamic studies. Dye. Pigment..

[43] Lafi R., Hafiane A. (2016). Removal of methyl orange (MO) from aqueous solution using cationic surfactants modified coffee waste (MCWs). J. Taiwan Inst. Chem. Eng..

[44] Yang Q., Wang Y., Wang J., Liu F., Hu N., Pei H., Yang W., Li Z., Suo Y., Wang J. (2018). High effective adsorption/removal of illegal food dyes from contaminated aqueous solution by Zr-MOFs (UiO-67). Food Chem..

[45] Jian M., Liu B., Zhang G., Liu R., Zhang X. (2015). Adsorptive removal of arsenic from aqueous solution by zeolitic imidazolate framework-8 (ZIF-8) nanoparticles. Colloids Surf. A Physicochem. Eng. Asp..

[46] Lin S., Song Z., Che G., Ren A., Li P., Liu C., Zhang J. (2014). Adsorption behavior of metal–organic frameworks for methylene blue from aqueous solution. Microporous Mesoporous Mater..

[47] Qiu T., Zeng Y., Ye C., Tian H. (2012). Adsorption Thermodynamics and Kinetics of p-Xylene on Activated Carbon. J. Chem. Eng. Data.

[48] Kuo C.-Y., Wu C.-H., Wu J.-Y. (2008). Adsorption of direct dyes from aqueous solutions by carbon nanotubes: Determination of equilibrium, kinetics and thermodynamics parameters. J. Colloid Interface Sci..

[49] Zhu X., Li B., Yang J., Li Y., Zhao W., Shi J., Gu J. (2015). Effective adsorption and enhanced removal of organophosphorus pesticides from aqueous solution by Zr-based MOFs of UiO-67. ACS Appl. Mater. Interfaces.

[50] Cavka J.H., Jakobsen S., Olsbye U., Guillou N., Lamberti C., Bordiga S., Lillerud K.P. (2008). A New Zirconium Inorganic Building Brick Forming Metal Organic Frameworks with Exceptional Stability. J. Am. Chem. Soc..

[51] Ai L., Zhang C., Liao F., Wang Y., Li M., Meng L., Jiang J. (2011). Removal of methylene blue from aqueous solution with magnetite loaded multi-wall carbon nanotube: Kinetic, isotherm and mechanism analysis. J. Hazard. Mater..

[52] Xiong L., Yang Y., Mai J., Sun W., Zhang C., Wei D., Chen Q., Ni J. (2010). Adsorption behavior of methylene blue onto titanate nanotubes. Chem. Eng. J..

[53] Wei C., Feng D., Xia Y. (2016). Fast adsorption and removal of 2-methyl-4-chlorophenoxy acetic acid from aqueous solution with amine functionalized zirconium metal–organic framework. RSC Adv..

[54] Wu S., Ge Y., Wang Y., Chen X., Li F., Xuan H., Li X. (2018). Adsorption of Cr(VI) on nano Uio-66-NH_2_ MOFs in water. Environ. Technol..

[55] Chizallet C., Lazare S., Bazer-Bachi D., Bonnier F., Lecocq V., Soyer E., Quoineaud A.-A., Bats N. (2010). Catalysis of Transesterification by a Nonfunctionalized Metal−Organic Framework: Acido-Basicity at the External Surface of ZIF-8 Probed by FTIR and ab Initio Calculations. J. Am. Chem. Soc..

[56] Fu J., Chen Z., Wang M., Liu S., Zhang J., Zhang J., Han R., Xu Q. (2015). Adsorption of methylene blue by a high-efficiency adsorbent (polydopamine microspheres): Kinetics, isotherm, thermodynamics and mechanism analysis. Chem. Eng. J..

